# The Role of the Inflammatory Response in Mediating Functional Recovery Following Composite Tissue Injuries

**DOI:** 10.3390/ijms222413552

**Published:** 2021-12-17

**Authors:** Naveena B. Janakiram, Michael S. Valerio, Stephen M. Goldman, Christopher L. Dearth

**Affiliations:** 1 Research & Surveillance Division, DoD-VA Extremity Trauma and Amputation Center of Excellence, Bethesda, MD 20889, USA; naveena.janakiram@usuhs.edu (N.B.J.); michael.valerio@usuhs.edu (M.S.V.); stephen.goldman@usuhs.edu (S.M.G.); 2 Department of Surgery, Uniformed Services University of the Health Sciences, Bethesda, MD 20889, USA; 3 Walter Reed National Military Medical Center, Bethesda, MD 20889, USA

**Keywords:** wound healing, tissue regeneration, inflammation, immunomodulation, military medicine, composite musculoskeletal trauma

## Abstract

Composite tissue injuries (CTI) are common among US Military Service members during combat operations, and carry a high potential of morbidity. Furthermore, CTI are often complicated due to an altered wound healing response, resulting in part from a dysregulation of the innate and adaptive immune responses. Unlike normal wound healing, in CTI, disruptions occur in innate immune responses, altering neutrophil functions, macrophage activation and polarization, further impacting the functions of T regulatory cells. Additionally, the biological underpinnings of these unfavorable wound healing conditions are multifactorial, including various processes, such as: ischemia, hypoxia, low nutrient levels, and altered cell metabolic pathways, among others, all of which are thought to trigger anergy in immune cells and destabilize adaptive immune responses. As a result, impaired wound healing is common in CTI. Herein, we review the altered innate and adaptive immune cells and their metabolic status and responses following CTI, and discuss the role a multi-pronged immunomodulatory approach may play in facilitating improved outcomes for afflicted patients.

## 1. Introduction

Composite tissue injuries (CTI) owing to high energy wounding mechanisms (e.g., explosions, gunshot wounds) are an unfortunate reality of modern military combat operations. In simple terms, CTI are those injuries that involve significant damage to multiple tissues, including skeletal muscle, nerve, bone, vasculature, and dermal tissues [1,2,3,4]. When such injuries affect the extremities, they often result in significant, long-term impairments in functional outcomes, and therefore delay or prevent the service member from returning to duty. As such, CTI are a critical concern of the Military Health System and a significant threat to the readiness of the joint force. Beyond military medicine, civilian populations are often impacted by CTI as a result of vehicular or industrial accidents [5], and these injuries represent a substantial economic burden in the order of billions of dollars annually (i.e., costs associated with hospitalizations, acute surgical services, long term rehabilitation costs, lost work hours) [6,7]. Surgical and rehabilitation costs, in particular, can be extraordinarily high owing to the inherent injury complexity and associated deleterious wound healing outcomes (e.g., delayed union, fibrosis); a key driver of such outcomes is the (dysregulated) endogenous immune-inflammatory response.

The immune-inflammatory response to acute injury is a highly complex system comprising multiple soluble (e.g., antibodies, complement) and cellular mediators (e.g., cytokines, prostaglandins) that serve to clear pathogens and cellular debris and stimulate tissue repair. Generally speaking, this system is highly redundant with multiple compensatory signaling pathways in place to ensure that the initial inciting stimulus is neutralized and that the injury progresses towards an end-stage wound healing outcome from both a histological and functional perspective. As with any complex system, however, substantial perturbations to the magnitude and/or spatiotemporal kinetics of this process—as is characteristic of CTI—can lead to impaired healing and a chronic pathology, even when wound healing is buttressed by potent exogenous therapeutics.

Exogenous therapies have demonstrated a limited benefit towards wound healing in CTI due to knowledge gaps concerning how best to modulate this dysregulated immune response as a means to promote improved wound healing outcomes. Therefore, a deeper understanding of the disrupted immune response following CTI will help to facilitate the development of next-generation, rationally informed therapeutic strategies which aim to facilitate improvements in wound healing with functional recovery. To that end, the disrupted inflammation and immune response resulting from CTI, and the associated interactions within the site of injury, are broadly discussed herein as a means to highlight potential targets for immunomodulation to enhance wound healing.

## 2. An Immune Mediated Inflammatory Response to Injury

**Figure 1:**
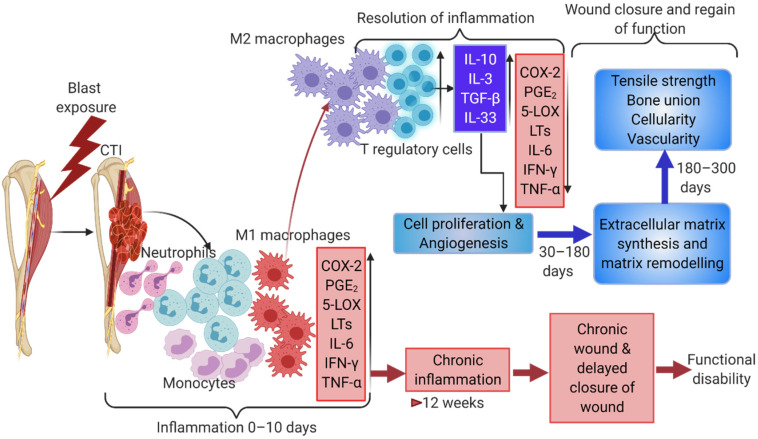
Composite tissue injuries involves multiple immune cells and endogenous immune pathways. Tissue-resident macrophages release chemoattractants triggering neutrophil infiltration and the simultaneous activation of complement pathway molecules in vessels. These events result in increased pro-inflammatory conditions with an increased number of M1-like macrophages. Pro-inflammatory arachidonic acid pathway metabolites, PGE2 and leukotrienes along with pro-inflammatory cytokines result in initiating precursor cells in various damaged tissues. An increase in Th2 immune response is established with an increase in IL10, IL4, and IL3, etc., which propagates M2 macrophages and Tregs that help in inducing cell differentiation and wound healing. A sustained pro-inflammatory reaction for a longer duration than needed results in a chronic wound or delayed wound closure.

Inflammation is a fundamental physiological response to injury. Acute injury leads to a cascade of inflammation-related events consisting of four overlapping phases: hemostasis, an inflammatory phase (involves resolution), a proliferation phase, and a remodeling phase [8]. During the inflammatory phase, the complement system initiates the infiltration of immune cells to the injury site [9]. Damaged cells release damage-associated molecular patterns (DAMPs) and trigger the secretion of attractant chemokines by resident macrophages [10,11]. Neutrophils rapidly infiltrate the wound and secrete oxidants and proteases to enable phagocytosis of damaged cells and tissues [12,13] (Figure 1). The longevity of neutrophils’ presence within the site of injury and the tight regulation of their function are required for a smooth transition to the proliferation phase of wound healing. For example, neutrophil-mediated production of inflammatory and chemotactic mediators, such as interleukin (IL)-1, IL-8, IL-6, and CCL2, (chemokine ligand 2 or monocyte chemoattractant protein (MCP)-1), initiate the second wave of inflammatory response, including the stimulation of resident macrophages [13,14] (Figure 1). Activated resident macrophages trigger the extravasation of monocytes to the site of injury that further differentiate into macrophages to amplify the population of these local cells. Simultaneously, neutrophils support the additional recruitment of macrophages by upregulating MCP-1 and chemokine ligand 3 (CCL3) [15]. Once within the injured tissue, macrophages secrete a number of soluble factors, including tumor necrosis factor-alpha (TNF-α), IL-1β, IL-6, and CCL2, which initiate the recruitment of a variety of cell populations, including fibroblasts, mesenchymal stem cells (MSCs), and progenitor cells. Macrophages at this stage function in a pro-inflammatory manner. These cells have been observed to reach peak numbers 1–2 days after injury [16,17] and produce robust inflammatory, chemotactic mediators and growth factors that govern the proliferation of various cell populations (e.g., fibroblasts, MSCs) and the deposition of the extracellular matrix.

Concurrently, but with slower kinetics, the adaptive immune system is likewise activated. The innate and adaptive immune responses exhibit an interdependent relationship wherein cross-talk is observed to regulate the complex series of events that facilitate wound healing. T lymphocytes are part of the adaptive cell-mediated immune response and play an important role in this cross-talk, both as producers of growth factors and through differentiation into effector cells. T lymphocytes are categorized into subsets based on functionality, with cytotoxic T lymphocytes—identified by the expression of CD8 surface markers—and helper T lymphocytes—identified by the expression of the CD4 surface marker—being the highest-level bifurcation germane to the discussion herein. These cells typically infiltrate into wounds after those of the innate immune response (e.g., neutrophils and macrophages) and play seemingly opposing roles. Cytotoxic T cells are considered the early responders of the adaptive immune system in an injury, and play an important role in enhancing the innate immune responses through expression of interferon (IFN)-γ and transforming growth factor (TGF)-β1 [18], and by recruiting inflammatory monocytes through T cell receptor signaling and secretion of MCP-1 [19]. Helper T lymphocytes, however, produce cytokines—specifically, high levels of IFN-γ, TGF-β1, IL-10, and IL-17—and affect neighboring cells through paracrine signaling to help regulate collagen deposition within the injured tissue. Secretion of IL-10 by helper T cells, in particular, regulates innate immune responses by mediating the transition of the inflammatory response as it drives the functional characteristics of the macrophage population from a predominately pro-inflammatory phenotype towards one of remodeling and repair.

The transition of the inflammatory response is also associated with an increase in recruitment of regulatory T lymphocytes (Tregs). Tregs typically peak in numbers at 3–5 days post-injury, and regulate the repair of injured tissue by modulating the inflammatory response [20]. Tregs attenuate tissue injury by suppressing the activity of neutrophils and pro-inflammatory macrophages and promoting the differentiation of MSCs. Furthermore, as an overabundance of active conventional T cells is detrimental to wound healing, Tregs can dampen CD4^+^ and CD8^+^ T cells through the secretion of anti-inflammatory cytokines such as IL-10, TGF-β, and IL-35 [21]. Additional evidence of this role is depicted in studies wherein the absence of Tregs was shown to enhance pro-inflammatory macrophage activity and IFN-γ expression [22] and increase the accumulation of CD8^+^ T cells and fibrotic collagen deposition within the site of injury [23]. Thus, it is clear that Tregs are highly involved in immune regulatory functions, and that the communication which exists between immune cells and MSCs orchestrate wound healing in several tissues (Figure 1).

During the later stages of the wound healing process, Tregs regulate the differentiation of a variety of cell populations, such as (Pax7^+^) satellite cells in skeletal muscle by continuously suppressing excessive inflammatory T cell responses (i.e., Type 1) while concomitantly being permissive to anti-inflammatory (i.e., Type 2) activity [23]. A variety of soluble factors (e.g., IL-10, IL-4, TGF-β, and AMPK activity) coordinate to augment anti-inflammatory conditions by resolving the inflammation and to induce stem cells to committed lineages regulating tissue composition, thus helping in the wound healing process [24]. Anti-inflammatory macrophages regulate extracellular matrix remodeling (which involves myofibroblasts and IL-13) and the proliferation of granulocytes and blood vessels within the injured region [25]. During the resolution of the inflammation and proliferation phase, macrophages produce numerous growth factors, such as insulin-like growth factor 1 (IGF-1), platelet-derived growth factor (PDGF), and vascular endothelial growth factor α (VEGF-α), and additionally, macrophages repress host immune responses by expressing programmed cell death ligands 1 (PDL1) and 2 (PDL2), which initiate Treg signaling and trigger anti-inflammatory responses, ensuring an enduring wound repair process [26,27].

## 3. Dysregulation of the Immune-Inflammatory Response Associated with Composite Tissue Injuries Contributes to Poor Outcomes

### 3.1. Altered Innate Inflammatory Response to Composite Tissue Injuries

As discussed above, inflammation is the initial biologic response observed in any injury [28]. For a successful wound healing outcome, the arrival, magnitude (or concentration), and persistence time of cellular and soluble (e.g., cytokines, chemokines) components of the innate and adaptive immune responses must be precisely regulated so as to allow a resolution of inflammation and subsequently facilitate tissue repair. CTI, however, triggers a shift away from this normal homeostatic response, resulting in several alterations to the canonical inflammatory cascade, and leading to a dysregulated response. Furthermore, the magnitude of the inflammatory stimuli emanating from multiple tissues within a CTI overwhelms regulatory mechanisms and leads to chronic pathologies in CTI patients [29,30]. Adding to the complexity, each injured tissue type within a CTI responds distinctly to the immune process concerning both the unique function of resident and infiltrating cell populations, as well as the spatiotemporal dynamics of when these actions occur. Here, we discuss how various innate immune cells and their responses are altered in CTI conditions, and how this disrupted response affects the transition of the acute inflammatory phase to the resolution of inflammation.

**Figure 2:**
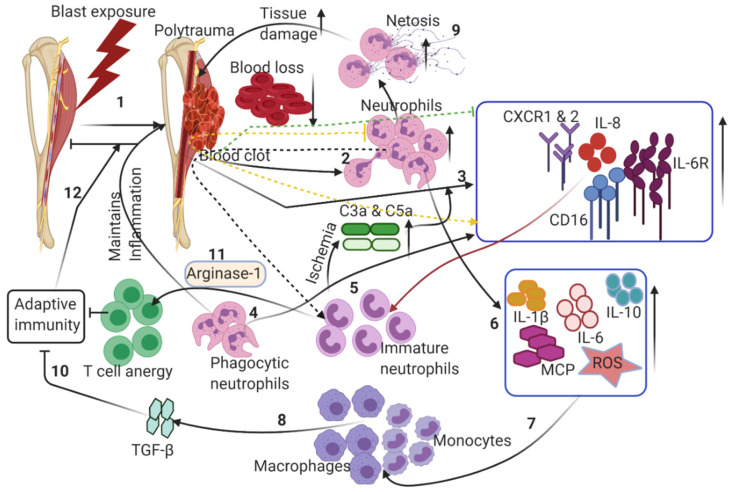
Dysregulation of innate immune responses following CTI: (1) blast-induced trauma in extremities damages multiple tissues, leading to hematoma. (2) Hematoma triggers neutrophil extravasation from the vasculature to the wound site. (3) A robust increase in pro-inflammatory cytokines and chemokines triggers neutrophils to clear the debris of dead cells. (4) Increased pro-inflammatory chemokines/interleukins induces the release of immature neutrophils, exacerbating the pro-inflammatory conditions. (5) An increase in immature neutrophils induces ischemia through the secretion of chemokines and proteases at the wound site. (6) Increased neutrophil infiltration further causes the upregulation of interleukins, chemokines and reactive oxygen species. (7) Monocytes and macrophages are activated to allow the transition from the acute inflammatory phase to the resolution phase. (8) Activated macrophages induce TGF-β. (9) Netosis of neutrophils induces robust pro-inflammatory responses, leading to excessive tissue damage. (10) Aberrant immune responses restrict the transition to adaptive immune responses. (11) Macrophages secrete arginase 1, thus inducing anergy in T cells. (12) These altered inflammatory responses maintain the presence of triggers for chronic inflammation, thus delaying the wound healing in CTI. Dotted arrows indicate the secondary induction of immune cells/cytokines, which inhibit normal healing responses by exacerbating pro-inflammatory responses. See text for further details.

Within minutes of tissue injury, gradual accumulation and coagulation of blood will naturally occur at the injury site, promptly forming a hematoma (Figure 2). As in the normal response to injury, hematoma and fibrin matrix formation signals an influx of inflammatory cells that are predominantly comprised of neutrophils [31]. Concomitantly, the complement system is activated and the production of various complement components is increased in correlation with the severity of the critical injury. It is apparent in CTI patients that these active components interact with their specific central membrane-bound and soluble complement regulatory proteins expressed on leukocytes (CD35, CD46, CD55, and CD59) and neutrophils (CD88) to trigger, exacerbate, and maintain the inflammatory reactions [32] (Table 1). Sufficient expression of surface receptors, including the IL-8 chemokine receptor (CXCR)1 and CXCR2, FcγRIII (CD16), IL-6 receptor (IL-6R), and complement receptor C5aR1, is required for the normal egress and active recruitment of neutrophils from the bone marrow to the injured tissue [15] (Table 1). Interestingly, the expression of these receptors is severely reduced in critical CTI injuries due to engulfment by neutrophils or release in macrovesicles [33,34]. IL-6 receptor (IL-6R) is continuously shed from the neutrophil surface, inducing increased inflammatory reactions (Table 1). Some published studies have reported that CTI typically induces functional changes in neutrophils, making them insensitive to persisting potential dangers [35,36]. Stepien et al. 2020 reported the prolonged presence of neutrophils and monocytes in a muscle injury and ischemia model, which exhibited a dampened wound healing response, reiterating the role of persistent pro-inflammatory conditions disrupting the necessary resolution of inflammation and regenerative regulatory pathways in wound healing [37]. Usually, neutrophil accumulation is mediated by chemo-attractants, such as C-X-C motif ligand (CXCL) 1, macrophage inflammatory protein-1α, the anaphylatoxin C5a, leukotriene B4 (LTB4), and IL-8 [13]. One of the harmful effects of neutrophils within a CTI is the uncontrolled release of IL-8, which mobilizes immature, less deformable neutrophils from the bone marrow, increasing inflammation and transcellular permeability, therefore compounding unfavorable conditions such as ischemia at the site of injury [38] (Table 1). The pre-existing ischemic environment, owing to disruption of the blood supply during injury, is exacerbated by the increased metabolic demand of the infiltrating immune cells in the zone of injury. Additionally, neutrophils degranulate and release free radicals, elastase, collagenase, and arachidonic acid to carefully clear the dead cells that accumulate (Table 1). This process typically triggers the induction of inflammatory response and increases edema and the shutting down of the local circulatory system, further exacerbating the ischemic condition [39].

Neutrophils express both intrinsic (naturally associated with mitochondria and B cell lymphoma-2-Bcl proteins) and extrinsic (promptly initiated by death ligand and death receptor) apoptotic pathways. It is interesting that the half-lives of pro-apoptotic genes are relatively longer than those of anti-apoptotic genes in these cells. The cellular levels of the anti-apoptotic gene-induced myeloid leukemia cell differentiation protein (Mcl-1) correlate with neutrophil survival and thus the half-life of neutrophils is governed by cellular levels of the anti-apoptotic gene Mcl-1 [40]. Pro-survival anti-apoptotic signaling pathways (e.g., Akt, PI3k, ERK, etc.) are activated to temporarily delay apoptosis to ensure the viability of neutrophils as they infiltrate the wounded region. Major trauma mostly consists of CTI-triggered anti-apoptotic genes that are transiently upregulated, increasing the persistence time of neutrophils in circulation [41]. Neutrophils instantly produce a significant amount of LTB4 and phagocytize damaged, dying cellular debris and subsequently may undergo NETosis, forming neutrophil extracellular traps (NETs) (Figure 2) (Table 1). These neutrophils likewise generate reactive oxygen species (ROS), antimicrobial peptides, serine proteases, and various cytokines and chemotaxins, including IL-1β, IL-6, IL-10, and MCP-1, driving macrophage/monocyte infiltrations [42,43] (Figure 2). The required mechanisms shown by neutrophils for the successful resolution of inflammation includes the clearance of cellular debris, and notably the production of anti-inflammatory cytokines (e.g., IL-10, IL-1rα, lipoxins, lysophosphatidylserine) to alter macrophage polarization for wound healing [44,45]. However, in a CTI, neutrophils are over-activated, leading to an altered cytokine profile, increased neutrophil heterogenicity [36], and an increase in NETosis, which aggravates tissue damage, leading to a toxic microenvironment (Table 1). A subtype of neutrophils, immunosuppressive low-density neutrophils (LDNs), observed in the thoraco-abdominal porcine trauma surgery [46], play an important role in suppressing adaptive immune responses by severely inhibiting T cell functions [47]. LDNs are not only activated, but also express high levels of arginase-1 activity, which regulates T cell function and thus impairs adaptive immunity in trauma patients afflicted with CTI and delays the successful transition to the proliferative phase of wound healing [48] (Figure 2) (Table 1). Neutrophils, however, also display certain beneficial effects which help in repairing the initial trauma wound. For example, neutrophils also secrete VEGF, initiating the angiogenesis process to facilitate blood circulation and wound healing [49]. Mostly, neutrophil functions are typically limited to the inflammatory phase of the wound healing unless prolonged persistent inflammation due to tissue degradation exists at the wound site, inhibiting an effective wound healing process. It is very clear in the little available literature that neutrophils play a vital role in initial immune responses after CTI and their functions largely affect wound healing responses. However, further in-depth research is necessary to elucidate the neutrophil associated with complications in trauma.

Macrophages and T cells from different tissues interact to relay required immune responses for enhanced wound healing. Other important innate immune cells that bridge between innate and adaptive immune responses are invariant NKT (iNKT) cells, a specific subtype of T cells, which are often neglected or not studied much in CTI wound healing. Other investigations on iNKT cells also indicate that iNKT cells play a vital role in wound healing [50,51]. Jo et al. reported fewer numbers of circulating iNKT cells in trauma patients compared to healthy patients [52]. The number of iNKT cells is negatively correlated with the severity of trauma observed in these patients with impaired proliferative responses [51,52]. It is speculated that increased levels of pro-inflammatory cytokines such as IL-6, IL-8, and TNF-α and decreased expression of IFN-γ in trauma patients might have caused the dysfunctional proliferation of iNKT cells [53]. iNKT cell-induced IFN-γ may regulate wound healing responses. IFN-γ interacts with myogenic precursor cells targeting JAK-STAT pathways, holding the macrophages at a proliferating state to initiate and support the proliferation process [54]. That said, the absence of iNKT cells and IFN-γ may delay wound healing. However, increased IFN-γ signaling in CTI inhibits angiogenesis and re-epithelialization, and it also inhibits collagen formation by antagonizing TGFβ signaling in wound repair. Hence, fine-tuned regulation of IFN-γ signaling is required to switch to the proliferative phase of wound healing.

As the necessary clearance of DAMPs occurs, neutrophils apoptose and are cleared by macrophages, therefore transmitting a detectable signal for the release of TGFβ1 and IL-10 by macrophages polarized by the cytokines IL-4, IL-13, and IL-10, promptly initiating a proliferative phase in wound healing. As the environment shifts away from a pro-inflammatory profile, fibroblasts proliferate to an active form of myofibroblasts in muscle tissue injuries. Selective TGFβ1 activation initiates the differentiation of myofibroblasts, which contribute positively to collagen synthesis. Fibroblasts play a role in skeletal muscle extracellular matrix formation and modulation in severe injury with potential loss of muscle mass [55]. Malecova et al. identified two types of fibroblasts based on the expression profiles of angiopoietin-2 receptor (Tie2) and vascular cell adhesion protein 1 (Vcam-1) within developing and injured muscle. The presence of Vacm-1 expression indicates a shift towards a fibrotic response. Increased Tie2 expression is observed in an uninjured muscle [56]. They convincingly demonstrated that the disruption of inflammation drives Vcam-1-expressing fibroblasts towards a fibrotic pathogenic outcome in injury. Recently, Stepien et al. 2000 observed an increased population and a shift in Tie2-positive fibroblasts to Vcam-1-positive fibroblasts in a profibrotic ischemia-reperfusion and muscle injury model [37]. Myeloid- (macrophage (Adgre1)), dendritic cell-, and granulocyte (Csf3r)-specific TGFβ1 deletion restored rapid muscle regeneration and the more rapid resolution of inflammation. Further experimentation with TGFBRII-Fc ligand trap mitigated muscular fibrosis, suggesting that this growth factor can properly regulate the complex immune response and can modulate fibroblast function towards fibrosis or regeneration during muscle repair.

The resolution of inflammation typically favors the production of collagen and the contraction of the lesion. Andermahr et al. made an insightful observation that in polytrauma patients—both with and without concurrent traumatic brain injury—an increase in collagen degradation resulted in decreased osteogenesis and non-union fractures [57]. They reported that pyridinoline cross-linked carboxy-terminal telopeptide (1CTP) was significantly higher in polytrauma patients. ICTP is secreted in response to collagen breakdown, collagen synthesis and breakdown by-products, and is detected in the blood during bone synthesis. Data indicate that in polytrauma, a lengthy pro-inflammatory condition prevails, disrupting the number and activity of fibroblasts, resulting in decreased collagen synthesis, thus resulting in a delayed union. This study is referenced here because of its significant findings in polytrauma with bone fractures which are often associated with CTI. Additionally, the role of fibroblasts is critical, since its deficit will slow down wound closure, or its uncontrolled activity can lead to excessive collagen deposition that may lead to fibrotic healing with impaired regeneration. This suggests that fibroblast proliferation and activity may decrease due to prolonged inflammation associated with CTI. Additionally, fibroblasts depend on anti-inflammatory macrophages, traditionally associated with non-chronic wounds. Both pro-and anti-inflammatory cytokine signaling interacts positively with myogenic and osteogenic regulatory pathways, in which an appropriate temporal conversion from type-1 (M1/Th1) to type-2 (M2/Th2) wound healing phenotypes is required for optimal regeneration. Along with anti-inflammatory macrophages, there is an independent subset of monocytes/macrophages, reported in CTI patients whose functions in wound healing are yet to be adequately investigated. West et al. (2012) showed an upregulation of TGF-β and macrophage colony stimulating factor (M-CSF) after a pro-inflammatory phase in CTI patients that helped in the differentiation of CD14^hi^CD16^+^, monocytes/macrophages, activated through C-reactive protein [58]. They also reported that the presence of the CD163 receptor on these cells indicated an alternative activation mechanism and that CD163 induces anti-inflammatory cytokines such as IL-10 in these cells. Induction of this specific type of myeloid population may enhance the anti-inflammatory environment, driving the wound healing regenerative phase. In a CTI, the integrity of surrounding tissue influences the rate of fracture healing. Severe traumas will lead to fractures along with extensive muscle tissue damage, delaying fracture healing. The profound increase in inflammation within an injured muscle can directly influence the immune cell infiltration and selective activation within the adjacent fracture defect. At the fracture site, bone-specific osteal macrophages also play a role in fracture healing, as they are localized close/next to mature osteoblasts and adequately provide support in bone healing, [59,60,61]. In vitro and in vivo removal of osteal macrophages from osteoblasts resulted in reduced osteoblast mineralization, supporting its role in bone formation [60,61]. Furthermore, osteoclasts originating from monocyte/macrophage precursors show increased activity during bony callus formation, and osteoclast bone resorption of the callus is followed by osteoblast-mediated bone formation [62]. TGF-β also helps in osteoblast differentiation and proliferation. Increased expression of TGF-β by monocytes observed in immunosuppressed patients indicate the presence of high levels of monocyte-derived TGF-β in blunt trauma patients [58]. Higher levels of TGF-β lead to an increased production of prostaglandin-E2 that is known to suppress adaptive T cell functions [63]. Therefore, this sufficiently indicates that elevated TGF-β levels by monocytes may impair adaptive T cell functions, which are needed for the successful proliferation and repair phases. It is apparent that innate immune responses naturally drive adaptive immune responses for the long-lasting effects.

### 3.2. Disruptions of the Adaptive Immune System in Composite Tissue Injuries

T-cell populations are diverse and secrete various cytokines and proteins to carefully regulate the proliferation and repair processes in a wound. Tregs, a specialized subpopulation of T cells which typically contribute towards balancing of Th-1 type pro-inflammation and Th-2 type anti-inflammation during the process of wound healing, are mostly prevalent in high numbers during the proliferative phase. Several published reports suggest that the presence of Tregs could confer faster wound healing [64] and may provide a survival advantage in CTI patients. Their increased number and activity were detected immediately after trauma, however, which resulted in an undesirable suppression of pro-inflammatory Th-1 type cytokine activities and lead to immune suppression and delayed wound healing [65]. Tregs attenuate the pro-inflammatory responses of monocytes [65] and regulate monocytes through the secretion of cytokines IL-10, IL-13, and IL-4. A marked increase in IL-10 concentrations was observed in trauma patients, indicating its role in Th1/Th2 shift mediated by Tregs [66]. This early increase in the expression of IL-10 with CTI could undoubtedly affect the healing process by suppressing vital pro-inflammatory responses. Moreover, Treg-derived IL-10 has a positive role in chondrocyte proliferation and differentiation in bone fractures. These roles are highlighted by observations that the loss of IL-10 in mice resulted in smaller proliferating zones in bones [67]. Mouse bone marrow-derived MSCs possess immunosuppressive properties. They are capable of shifting from a pro- to anti-inflammatory phenotype, resulting in decreased production of pro-inflammatory cytokines, mediated by TNF-α stimulated gene/protein 6 (TSG-6) and prostaglandin E-2 [68,69,70]. Treg-derived IL-10 inhibits T cells and specifically suppresses Th-1-type cells and their pro-inflammatory cytokines, such as IFN-γ. Tregs also play an important role in controlling/extending pro-inflammatory conditions. There is a possibility that in the presence of TGF-β and IL-6, these cells are susceptible to being efficiently converted into the pro-inflammatory Th-17 cells [71]. Therefore, this naturally raises the need to properly understand the altered cytokine profiles which may exist in CTI that lead to the activation of Tregs during initial phases/immediately after trauma. It is known that Tregs derived from naïve CD4^+^ T cells positive for Foxp3^+^ and CTLA4^+^ suppressed the proliferation of NKT and CD4 and CD8 T cells through contact inhibition facilitated by CTLA4 and CD36 receptors [72]. Tregs mitigate this function through immature dendritic cells (DC), which express low levels of co-stimulatory molecules. Tregs rigorously suppress the maturation of DCs, thus inducing anergy in T cells [73]. Tregs also regulate the macrophage phenotype and functions [74]. Tregs suppress neutrophils and conventional T cell functions to initiate the transition of the inflammatory phase to the regenerative and repair phase. Loss of Treg functions in a CTI might impair this transition. Co-culture of Tregs with muscle satellite cells enhanced the expansion of satellite cells, indicating cell to cell interaction [75]. This suggests that Tregs not only regulate other immune cells through the secretion of cytokines but also through cell–cell interactions. Furthermore, the absence of Tregs diminished myogenic activity in the acute injury of skeletal muscle [23]. These reports sufficiently emphasize the role of Tregs in the proliferative/regenerative phase of wound repair. In muscle and adipose tissue, Tregs express amphiregulin (Areg), an epidermal growth factor (EGF)-like growth factor, involved in controlling muscle-homeostasis, function and repair. The administration of Areg decreased the expression of genes encoding proteins related to fibrosis (e.g., a battery of collagens, Adam12, Acta2), and enhanced the expression of gene encoding molecules, such as Pfkfb1 and 3 and Myl2, highly represented in the healthy muscle [23]. Additionally, Areg promoted increased myogenic differentiation of satellite cells, and these cells expressed high levels of transcripts and protein of myosin heavy chain. Furthermore, emphasizing the significance of this pathway, accumulation, and functions of injury-associated-Tregs that are stimulated by IL-33. Loss of IL-33 in stimulated Tregs resulted in impaired tissue repair [23]. Very little information exists on how amphiregulin regulates the Treg functions under CTI conditions presently. What is known about Tregs’ altered functions in CTI is listed in Table 2. Research into the regulatory effects of the various growth factors on Tregs within the context of CTI represents an opportunity for further research, as knowledge is currently very limited at this stage.

### 3.3. Deleterious Effects of Immune-Inflammatory Dysregulation on Wound Healing

Wound healing involves immune cells, cytokines, growth factors, and progenitor cells, such as FAPs, which are mesenchymal in origin [76]. The physiologic response to an injury triggers fibrogenic or adipogenic differentiation of FAPs in an immune-dependent (i.e., IL13 and IL4) fashion [76]. During the proliferation phase, IL-4/IL-13 signaling via signal transducer and activator of transcription 6 suppresses the differentiation of FAPs into adipocytes and generates tissue-specific cells supporting wound healing. Immediately after injury, an acute repair response is triggered, which heals the damaged tissues, whereas in a CTI, a heightened inflammatory response leads to the death of cells and the infiltration of immune cells. A transition from pro-inflammatory to anti-inflammatory immune cells drives the proliferation phase, which is a critical step for successful wound repair. A prolonged inflammatory milieu of more than 3 days after injury in CTI disturbs the well-orchestrated cellular choreography between inflammatory, FAP, and progenitor cells, resulting in the fibrotic and adipogenic degeneration of injured tissue, with an end result of an impaired function of the afflicted tissues.

## 4. Consequences of an Altered Wound Environment on Metabolic Processes and Downstream Effects on Inflammatory Signaling Pathways

**Figure 3:**
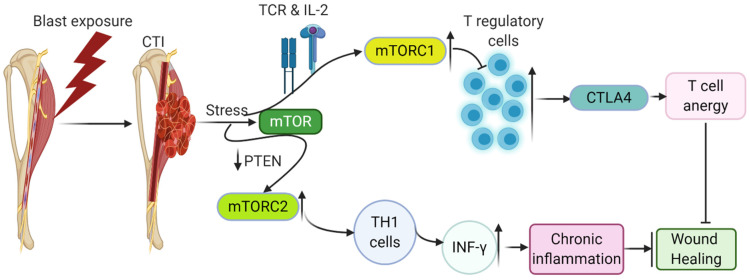
Changes in the mTOR metabolic pathway due to stress created by CTI triggers anergy in T cells, delaying adaptive immune responses by supporting the induction of pro-inflammatory cytokines, leading to chronic inflammatory conditions and delay/inhibit wound healing.

In CTI, hypoxic conditions, edema, low nutrient levels, and contributing factors downstream of cell metabolic pathways effectively stimulate immune and vascular endothelial cells to release various cytokines and growth factors that impair angiogenesis and regeneration. If neovascularization is successful, it allows the possible restoration of nutrient delivery and oxygen, and cells use oxidative metabolism for their longer-term functions and contribute positively to restoring the wound. Otherwise, the cells undergo stressful conditions, leading to the release of altered metabolic stressors and sensors. The mammalian target of rapamycin (mTOR), general control nonderepressible 2 (GCN2) kinase, hypoxic conditions, and adenosine, the metabolite of ATP, are known metabolic regulators and sensors that cause anergy in immune cells [77]. These pathways regulate the induction of anergy, ensure the maintenance of anergy in T cells, and sense/respond to the presence of hypoxia, changing nutrient levels and modulating metabolic processes, including glucose, lipid, energy, and metabolism. These metabolic pathways play a role in switching catabolic and anabolic pathways in T cells, regulating their activation, proliferation, and differentiation. T-cell receptor (TCR) and IL-2 promote the mTORC1 complex of mTOR and suppress Tregs, enhancing effector T-cell molecules such as CTLA-4, which can cause anergy in T cells, as discussed above. Under inflammatory conditions, over activation of mTORC1 leads to a loss of Tregs stability and the conversion of Tregs to effector T cells. Effector T cells producing cytokines such as IL-17 and IL-1β create a pro-inflammatory environment [78]. An increased mTORC2 complex of mTOR activation due to the deficiency of phosphatase and tensin homolog (PTEN) enhances IFN-γ-producing Th1 cells [79] (Figure 3). PTEN is a negative regulator of the phosphoinositide 3-kinase (PI3K) pathway. Therefore, increased mTORC2 activity destabilizes Tregs and alters Tregs-mediated inhibition of Th1 cells. Hence, optimum mTOR activity is required to restore the proper functions of Tregs. The disturbed environment in a CTI environment by releasing various proteins such as semaphorins, which act as axon guidance factors of the developing nervous system, may interact with Tregs-expressed receptor neuropilin-1, thus increasing the activity of PTEN [80], impairing mTOR signaling. Semaphorins are detected in the fibroblasts at the injury site that will regulate the neuronal proliferation after injury (Figure 3).

**Figure 4:**
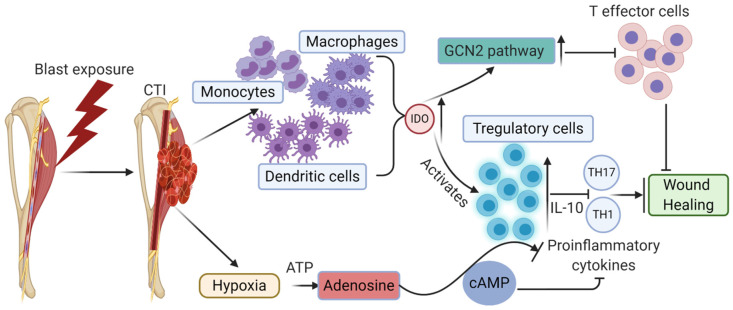
Stress-induced amino acid-sensitive pathway, GCN2 signaling is activated in CTI conditions at the very early stages, thus inducing Treg functions not necessary at that stage of wound healing, thus inhibiting/disrupting the natural course of the acute pro-inflammatory phase. Increased expression of Tregs at early stages delays wound healing through the GCN2 pathway.

A second amino acid-sensitive pathway, GCN2 signaling, also affects immune responses under stress conditions. GCN2 signaling can trigger stress response programs causing cell differentiation, suppression, growth arrest, apoptosis, and adaptation to stressful conditions [81,82]. GCN2 acts as a metabolic sensor in T cells in response to indoleamine 2,3-dioxygenase (IDO)-triggered conditions [83] (Figure 4). IDO is a metabolic pathway involved in tryptophan degradation in the kynurenine pathway. IDO controls the molecular switch between pro-inflammatory effector T cells and suppressor Tregs during inflammation. In severe trauma, increased IDO activity was observed in patients as an essential part of the defense mechanism by the body [84,85]. IDO is highly expressed in monocytes, macrophages, DCs, which upregulates the GCN2 pathway, decreasing T-cell priming, T-cell effector functions, and T-cell proliferation. On the other hand, high IDO increases Tregs activation and suppresses Th1 and Th17 cytokines and other pro-inflammatory cytokines (e.g., IFN-γ, TNF-α, and IL-2), driving Tregs function towards the resolution of inflammation during wound healing (Figure 4). TNF signaling represses stem cell proliferation through the p38-MAPK signaling pathway, thus regulating myogenesis in tissue remodeling and repair processes [24,86]. However, chronic IDO activity may be detrimental at the initial inflammatory phase of wound healing. Although we have a mechanistic understanding of how Tregs are modulated under various stressful environmental conditions, it is important to unravel the responsible mechanisms involved in enhancing the number of Tregs at the transition of the resolution phase, which fares better than a smaller number of Tregs at this stage in wound healing.

Tregs also have shown a complementary immunological arm under unfavorable conditions such as hypoxia. Hypoxia is generally observed in CTI patients due to low oxygen conditions and an inflamed microenvironment. Nanobashvili et al. (2003) reported that 51% of the analyzed patient population showed distal ischemia/low oxygen levels due to mangled injuries associated with damaged vessels [87]. Nitecki et al. reported limb ischemia in 75% of CTI patients, who mostly had extremity vascular injuries [88]. These data suggest the apparent prevalence of hypoxia/ischemia in CTI. Post-trauma edema further reduces the oxygen supply to the damaged tissues, thus impairing the neoangiogenesis and required resupply of nutrients for regeneration and wound healing. The hypoxic microenvironment formed due to a lack of oxygen and nutrients drives the cells to produce adenosine from extracellular ATP. The immunosuppressive extracellular metabolite adenosine typically plays an immune regulatory role in Tregs. Ectonucleotidases CD39 and CD73 degrade ATP to produce adenosine. CD39 and CD73 are immune checkpoint mediators expressed on Tregs [89]. A2aR and A2bR adenosine receptors stimulate intracellular adenylyl cyclase to synthesize cAMP. A2aR and A2bR adenosine receptors are present on T cells. Increased levels of cAMP lead to immunosuppressive effects, such as a decrease in pro-inflammatory cytokine IFN-γ and an increase in the production of anti-inflammatory cytokines such as IL-10 and TGF-β. Adenosine produced by Tregs interacts with other immune-infiltrating cells through A2aR and A2bR adenosine receptors, thus activating or inactivating the other immune cells [90]. Prolonged hypoxic conditions at the early stages of CTI may trigger Tregs responses at very early phases, disturbing the required pro-inflammatory cytokine profile and thus hampering the wound healing process. A deeper understanding of this phenomenon in CTI can provide us with possible clues for modifying the environmental conditions and immune responses for a faster-desired wound healing process.

## 5. A Multi-Pronged Immunomodulatory Strategy as a Potential Opportunity to Facilitate Improved Outcomes Following Composite Tissue Injuries

In the above sections, a detailed description of how the immune-inflammatory response interacts throughout both the normative physiologic and CTI-related pathophysiologic wound healing processes was provided as a means to provide a foundation upon which next-generation, rationally informed, multi-pronged immunomodulatory strategies can be developed as a means to facilitate improvements in outcomes following CTI. Furthermore, the spatiotemporal application of these therapies should be conducted in coordination with the known pathophysiology of CTI injury. Thus, a multi-pronged immunomodulatory strategy is an attractive option to complement existing CTI treatment strategies.

We previously discussed in detail how uncoordinated interactions between various immune cells at the injury site contribute to a highly unsynchronized microenvironment resulting in impaired wound healing. An example of how immune cells can be modulated to coordinate successful wound healing is provided by macrophages, in which staggered bolus release of IFN-γ and subsequent sustained release of IL-4 promote a shift from a pro-inflammatory to anti-inflammatory phenotype to mediate the scaffold vascularization [91]. Pro-resolving mediators such as lipoxins and resolvins have shown their inhibitory effects on neutrophils and macrophages. These mediators may have a benefit in CTI to reduce pro-inflammatory effects of neutrophils and macrophages diverting towards an anti-inflammatory response and regeneration of wound [92,93]. Implantation of chitosan scaffolds with lipoxins and resolvins in a mouse model polarized macrophages towards an anti-inflammatory phenotype and subsequently reduced fibrosis [92,93]. TGF-β is another potent molecule that has both anti- and pro-inflammatory functions, and caution is advised in wound repair if using this molecule. TGF-β has been reported to induce Tregs, which drive the regeneration process during wound healing [94]. A positive action of TGF-β3 in decreasing post-operative scar formation in a clinical trial is encouraging, and also its use in the CTI model [95,96]. The action and interplay between pro-inflammatory and anti-inflammatory for positive wound regeneration and specifically the effects of timing still need work.

Further revelation of the regulatory components involved in the development of inflammatory reaction to CTI holds great potential in the development of effective treatment strategies to maximize immune elements critical for repair, while at the same time suppressing elements of the immune response responsible for further damage to injury progression.

## 6. Conclusions

Composite tissue injuries remain a major challenge to the US Military and pose the threat of lifelong disability for those affected. Achieving improved clinical outcomes of these injuries will require restoration of normative inflammation and immune cell interactions at the site of injury and redressment of the hallmark inflammatory abnormalities of CTI that disrupt normal wound healing mechanisms. Specifically, a prolonged or heightened inflammatory phase, dysregulation or activation of pro-inflammatory immunological cells/factors, unwanted anti-inflammatory microenvironment at the very early phase of wound healing, and undesired alteration in the cytokine profile at a very initial phase must be addressed to achieve such clinical goals. Additionally, other stress factors such as hypoxic conditions, ischemia, low nutrient levels and altered metabolic processes that influence the immune functions must be managed concurrently. As such, CTI treatment strategies should aim to precisely address the cytokine network dysregulation so as to modulate the secretory profile of various damaged tissues. Ostensibly, this can be achieved by modulating the cytokines themselves (e.g., exogenous supplementation) or by precisely controlling the complex spatiotemporal dynamics of different immune cell populations, of which neutrophils and Tregs seem to be promising targets. Practically, however, this is a foreboding task, as very little is known of how the initial inflammation following an injury interacts with local regenerative processes in the context of CTI, and much research is needed to improve our understanding to the point of identifying precise targets for therapeutics which will not have deleterious effects elsewhere in the wound healing process. Specifically, ambiguity still exists as to which phases of immune-inflammatory response are needed to promote successful wound healing outcomes, and the optimal timing for these phases to resolve. These factors are critical, as both insufficient inflammation and chronic inflammation may lead to impaired wound healing and/or scar formation. Therefore, more detailed experimental evidence is needed to help us accurately decide how we could carefully manipulate inflammatory responses towards wound healing and to make informed decisions on the dosing (e.g., magnitude, frequency, duration) of immunomodulatory agents following composite musculoskeletal trauma.

## Figures and Tables

**Table 1:** CTI induced alterations in neutrophils and their functional consequences.

Trauma Induced Changes in Neutrophils	Functional Consequences
Expression of CD88	Generation of superoxide anions and release of granule enzymes-helps in adhesion to endothelial cells.
Engulfment of IL-8 chemokine receptor (CXCR)1 and CXCR2, FcγRIII (CD16), and complement receptor C5aR1	Impairment of targeted chemotaxis causing inflammatory disorder
Active shedding of IL-6 receptor (IL-6R)	Amplify inflammatory effects
Uncontrolled release of IL-8	Mobilizes immature, less deformable neutrophils
De-granulate and release free radicals, elastase, collagenase, and arachidonic acid	Trigger inflammatory response, aggravate ischemia and shut down local circulatory system
Increase anti-apoptotic genes	Increase circulation half-life of neutrophils
Increase in NETosis	Aggravate tissue damage
Increase in Low-density neutrophils (LDNs)	Suppress adaptive immune responses

**Table 2:** T regs display both pro and anti-inflammatory responses in CTI during initial phases/immediately after trauma.

T regs in CTI	Functional Consequences
Increase in Tregs number and activity immediately after CTI	Undesirable suppression of pro-inflammatory Th-1 type cytokines delaying wound healing
Early phase increased expression of IL-10	Suppression of pro-inflammatory responses (Th1 type and IFN-γ delaying wound healing
Presence of TGF-β and IL-6 convert Tregs to TH17 cells	Increase unrequired inflammatory responses
T regs suppress DC maturation	Inducing anergy in T cells
T regs loose regulatory function on neutrophils, and conventional T cell functions	Loss of transition of inflammatory phase to regenerative and repair phase
Absence of Tregs	Myogenic activity
Expression of amphiregulin by Tregs	Controls muscle-homeostatis
Loss of IL-33 in stimulated Tregs	Impaired tissue repair

## Abbreviations

CTI	Composite tissue injuries
(DAMPs)	Damage-associated molecular patterns
TNF-α	Tumor necrosis factor-alpha
CCL2	Monocyte chemoattractant protein-1, MCP-1
IL	Interleukin
CCL3	Chemokine ligand 3
CD	Cluster of differentiation
IFN	Interferon
TGF	Transforming growth factor
Tregs	Regulatory T cells
AMPK 5′	Adenosine monophosphate-activated protein kinase
IGF-1	Insulin-like growth factor 1
PDGF	Platelet-derived growth factor
VEGF-α	Vascular endothelial growth factor α
PDL1	Programmed cell death ligands 1
FAP cells	Fibro-adipogenic progenitor cells
CXCR	Chemokine receptor
Bcl-2	B-cell lymphoma 2
MCL1	Induced myeloid leukemia cell differentiation protein
LTB4	Leukotriene 4
NETs	Neutrophil extracellular traps
ROS	Reactive oxygen species
MIP	Macrophage inflammatory protein-1α
LDNs	Low-density neutrophils
iNKT	Invariant NKT
Tie2	Angiopoietin-2 receptor
1CTP	Pyridinoline cross-linked carboxy-terminal telopeptide
MCSF-1	Macrophage colony-stimulating factor
DC	Dendritic cells
Areg	Amphiregulin
EGF	Epidermal growth factor
mTOR	Mammalian target of rapamycin
GCN2	General control nonderepressible 2
TCR	T-cell receptor
PTEN	Phosphatase and tensin homolog
PI3K	Phosphoinositide 3-kinase
IDO	Indoleamine 2,3-dioxygenase
